# The impact of storage buffer, DNA extraction method, and polymerase on microbial analysis

**DOI:** 10.1038/s41598-018-24573-y

**Published:** 2018-04-19

**Authors:** Luisa K. Hallmaier-Wacker, Simone Lueert, Christian Roos, Sascha Knauf

**Affiliations:** 1 Work Group Neglected Tropical Diseases, Infection Biology Unit, German Primate Center, Leibniz Institute for Primate Research, Kellnerweg 4, 37077 Göttingen, Germany; 2 Primate Genetics Laboratory, German Primate Center, Leibniz Institute for Primate Research, Kellnerweg 4, 37077 Göttingen, Germany

## Abstract

Next-generation sequencing approaches used to characterize microbial communities are subject to technical caveats that can lead to major distortion of acquired data. Determining the optimal sample handling protocol is essential to minimize the bias for different sample types. Using a mock community composed of 22 bacterial strains of even concentration, we studied a combination of handling conditions to determine the optimal conditions for swab material. Examining a combination of effects simulates the reality of handling environmental samples and may thus provide a better foundation for the standardization of protocols. We found that the choice of storage buffer and extraction kit affects the detected bacterial composition, while different 16S rRNA amplification methods only had a minor effect. All bacterial genera present in the mock community were identified with minimal levels of contamination independent of the choice of sample processing. Despite this, the observed bacterial profile for all tested conditions were significantly different from the expected abundance. This highlights the need for proper validation and standardization for each sample type using a mock community and blank control samples, to assess the bias in the protocol and reduce variation across the datasets.

## Introduction

Microorganisms colonize various anatomical sites and play a crucial role in the balance of health and disease. The vaginal microbiome is known to maintain the health of women and thereby prevents urogenital diseases^1^. The advent of cultivation-independent molecular approaches, such as 16S rRNA amplicon sequencing, has allowed for a better understanding of the microbes that inhabit different biological niches. However, these powerful tools are not without important technical caveats that can lead to a distortion in the acquired data^2^. Such limitations have been well documented, and include sample collection, storage buffer, DNA extraction, amplification primers and methods, sequencing technology, and analysis techniques^3,4^. While it is impossible to negate all of these influences, it is important to understand the bias inherent in the analysis. Studies focusing on one or two technical limitations have made recommendations for improving the bias such as reducing the number of PCR cycles^5^ or adding additional lysis pre-treatment^6^.

DNA extraction, a critical step in culture-independent bacterial profiling, has been identified as a key driver of technical variation^3^. Most common studies on the microbiome of swab material use commercially available DNA extraction kits that vary in their lysis approach from mechanical to enzymatic treatment. Various studies have focused on technical variations in extraction kits, yet a field-wide consensus on sample extraction has not been reached^3,6–9^. Due to the large variety of microbiota and sample types, a single standard for all sample types is unlikely to be achieved. Despite the knowledge that the choice of extraction kit can have a significant effect on the results, there is often a lack of proper validation across sample types^3^.

Similar to DNA extraction kits, the choice of sample storage buffer has been shown to influence the detected bacterial community^10–12^. The ideal storage choice largely depends on the available resources during sampling such as the availability of freezing conditions^11^. Selecting the optimal storage buffers is dependent upon its compatibility with all downstream analyses including the extraction method. Many studies, however, only focus on the effect of a single technical variation instead of examining the effect of different combination of storage buffer, DNA extraction kit, and amplification methods^2^. Studying a combination of effects mirrors the reality of sample handling more closely and may thus provide a better foundation for the standardization of sampling handling protocols prior to microbial analysis.

**Figure 1:**
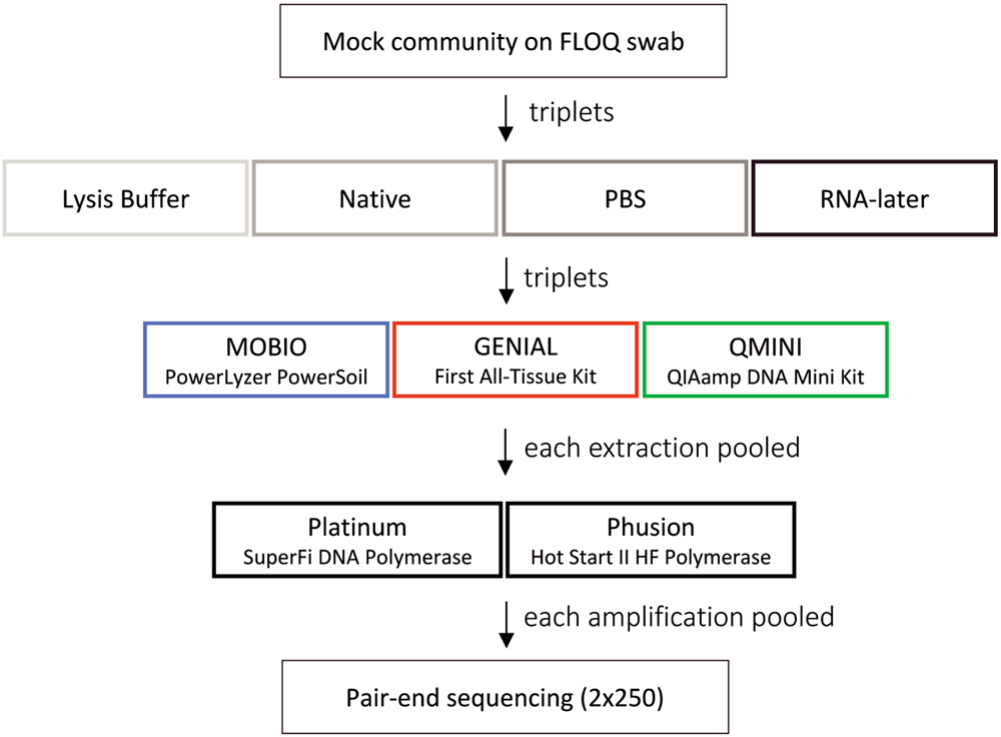
Outline of experimental design. A schematic showing the different treatment variables.

In this study, we used a mock community, composed of an even concentration of cells from 22 bacterial strains (19 genera), to assess the effect of storage buffers, extraction kits, and amplification methods (Fig. 1). Using a mock community to examine the effect of different sample handling conditions rather than environmental samples of unknown microbe composition is essential to be able to systematically compare the effects^3^. In addition to the use of a mock community, a blank control was included in all sample procedures to monitor any buffer, kit, or reagent specific contamination^13^. The aim of this study was to evaluate the performance of combinations of handling conditions commonly used in microbiome studies and to contribute to the ongoing debate on standardization in microbiome research.

## Methods

### Preparation of swab mock community samples

A cell mixture of 22 different bacterial strains at a concentration of 1 × 10^8^ cells/mL of each organism (Microbial mock community, HM-280) in phosphate buffer saline (PBS) was obtained through Biodefense and Emerging Infectious Research (BEI) Resources, NIAID, NIH as part of the Human Microbiome Project (Manassas, USA; Supplementary Table S1). To simulate physiological conditions, 10 μl of mock community containing 1 × 10^6^ cells/mL of each organism was added to a flocked swab (FLOQSwabs, Copan Improve Diagnostics, Brescia, Italy) and then placed in 500 μl of the respective storage buffer (Fig. 1). Four different storage buffers were used; PBS (PAN-Biotech GmbH, Aidenbach, Germany), a custom-made lysis buffer (10 mM Tris, pH 8.0, 0.1 M EDTA, pH 8.0 and 0.5% SDS), RNA-later (Thermo Fisher Scientific Inc., Waltham, MA, USA), and no buffer (native). A blank control swab sample was placed in each storage buffer without additive. All swab samples were frozen at −80 °C for one week prior to DNA extraction. Suitable precautions were taken during sample handling and processing to insure sterility during all procedures.

### DNA Extraction methods

Three commercially available DNA extraction kits were used in this study to extract bacterial DNA from swab material stored in four different storage buffers (Table 1). Extraction was performed in triplets and the extracted DNA from each buffer was subsequently pooled prior to 16 S rRNA gene amplification. Processing of swab samples prior to DNA extraction is illustrated in Supplementary Fig. S1.

**Table 1:** Commercial extraction kits used in this study.

Extraction Method	Abbreviation	Lot #	Lysis type	Elution Volume
MOBIO PowerLyzer PowerSoil Kit	MOBIO	PL16C30	Mechanical, Column-based	100
GEN-IAL First All-Tissue Kit	GENIAL	0091.01	Enzymatic, Phenol-Chloroform	20
QIAamp DNA Mini Kit	QMINI	154035749	Enzymatic, Column-based	70

### QIAamp DNA Mini Kit (QMINI)

Samples were extracted using the QIAamp Mini Kit (Qiagen GmbH, Hilden, Germany) according to the standard protocol with minor modifications. Briefly, proteinase K (20 mg/μl) was added and the samples were incubated for 50 minutes at 56 °C. Then, AL buffer (Qiagen GmbH) and ethanol were added in the appropriate amount. The DNA from the lysate was subsequently purified using the spin columns provided by the manufacturer and eluted in 70 μl AVE buffer (Qiagen GmbH).

### MOBIO PowerLyzer PowerSoil Kit (MOBIO)

A maximum of 750 μl of swab lysate was added to the 0.1 mm PowerLyzer® Glass Bead Tube (Qiagen GmbH). DNA extraction was continued from step 2 as described in the MOBIO PowerLyzer PowerSoil Kit protocol (Qiagen GmbH). The DNA was eluted in a final volume of 100 μl of Solution C6 provided in the kit.

### GEN-IAL First All-Tissue Kit (GENIAL)

The first All-Tissue Kit (GEN-IAL, Troisdorf, Germany) was applied according to the manufacturer’s protocol with minor modifications. Briefly, 5 μl proteinase K and 5 μl dithiothreitol (DTT) was added to the lysate and incubated at 65 °C for 60 min at 600 rpm in a thermomixer (Eppendorf, Hamburg, Germany). The lysate was purified according to the standard protocol and the DNA pellet was resuspended in 20 μl of C6 buffer (Qiagen GmbH).

### 16S rRNA gene amplification

For each pooled extraction, the V4 region of the 16 S ribosomal RNA (16 S rRNA) gene was amplified in triplets using the universal primers 515 F and 806 R adapted with linker regions and barcoded sequences used for dual-indexing^14^. Platinum SuperFi DNA Polymerase (Thermo Fisher Scientific) and the Phusion Hot Start II High-Fidelity DNA Polymerase (Thermo Fisher Scientific) were both tested for amplification. Each PCR reaction consisted of 12.5 μl of 2x PCR master mix, 6 μl of Microbial DNA-Free water (Qiagen GmbH), 1.25 μl of each primer (0.5 mM each, Metabion, Martinsried, Germany) and 4 μl of template in a total reaction volume of 25 μl. PCR cycling conditions comprised of a pre-denaturation step of 30 s at 98 °C, followed by 30 cycles of 98 °C for 10 s, 55 °C for 15 s and 72 °C for 60 s, and a final 10 min extension step at 72 °C. For a selection of four samples, five additional cycles were added to the amplification procedure to examine if additional cycles may be favorable for samples with low concentrations. The amplicon triplets were pooled, purified using 0.7x AMPure XP beads (Beckman Coulter, Brea, USA) and quantified using the Qubit 2.0 Fluorometer (Thermo Fisher Scientific). Amplicon integrity was verified for a representative number of 11 samples using a BioAnalyzer 2000 (Agilent, Palo Alto, USA) prior to pooling equimolar amounts (10 nM) of each amplicon for sequencing. For the blank samples, the maximum volume (5 μl) of sample was added to the library, as the concentrations prior to sequencing were below 10 nM. Illumina MiSeq. 2 × 250 bp paired-end sequencing (Illumina V2 chemistry) was performed in the Transcriptome and Genome Analysis Laboratory at the University of Göttingen^14^. All generated read files analyzed in this study were uploaded to the NCBI Sequence Read Archive (SRA) (SRP125723).

### Mock community data processing and analysis

The sequencing reads were processed using the mothur software package (v.1.36.1)^15^. According to the MiSeq SOP^14^, contigs were assembled, sequences trimmed, identical sequences merged, and chimeras removed (UCHIME^16^). Subsequently, sequences were aligned to the SILVA bacterial reference database^17^. Non-bacterial sequences, cross-sample singletons, and poorly aligned sequences were removed. The seq.error command was run for each mock sample in mothur and subsequently averaged to determine the error rate of the run. Due to low read numbers, blank control sample reads (control swabs containing no mock community) were removed from the dataset and analyzed separately. As subsampling is currently still an accepted method of normalization in microbial ecology^18^, the reads of the remaining mock community samples were rarefied to 95,870 sequences/sample. A separate file with the theoretical sequence composition (actual) of the 22 bacterial strains of mock community was created and adjusted for the 16 S rRNA copy number (Supplementary Table S1) and normalized to the sequence count of the run (95,870 reads)^19^. After merging the actual (theoretical) mock community composition with the practically obtained sequences, the merged file was classified using the Bayesian classifier implemented in mothur^20^. Operational taxonomic units (OTUs) were assigned based on 97% sequence similarity and subsequently the alpha and beta diversity was analyzed. For alpha diversity, the richness (OTUs observed and Choa1) and community diversity (Inverse Simpson Metrix) was analyzed using the summary.single command in mothur. Additionally, the percentage of contaminant OTUs (OTUs that do not cluster to the theoretical mock community) was examined. Beta diversity was analyzed using Bray-Curtis dissimilarity index^21^. The dissimilarity matrix was visualized using nonmetric multidimensional scaling (NMDS) plots and Newick formatted dendrograms (visualized in FigTree v.1.4.2, http://tree.bio.ed.ac.uk/software/figtree/).

### Statistical comparison of sequence data

To evaluate and compare the type of extraction and amplification method used, the values of the alpha or beta diversity measurement were pooled for each variable (e.g. the buffer type). The statistical significance of the pooled data was analyzed in GraphPad Prism 6 (GraphPad software, La Jolla, CA, USA). In case of normal distribution (Kolmogorov-Smirnov normality test), the parametric paired two-tailed students t-test was used for comparison. In all other cases the non-parametric Wilcoxon matched-pairs signed rank test was used. For multiple comparisons, a one-way ANOVA with Bonferroni’s multiple comparisons test was applied. Differences in community structure between storage buffers and extraction methods were tested using analysis of molecular variance (AMOVA) in mothur^22^. Non-metric multidimensional scaling (NMDS) plot of Bray-Curtis dissimilarities and UPGMA-clustered dendrograms (Bray-Curtis) were used to visualize data points. Parsimony (mothur) hypothesis testing was performed to test whether the differential clustering of the PBS samples in the dendrograms was significant^23^. Differences in the 30 most abundant OTUs were assessed using the metastats command in mothur^24^ and p-values for differences in individual OTUs were corrected for multiple comparisons using Bonferroni correction. Values of p < 0.05 were considered statistically significant.

## Results

The pooled library (n = 28 mock samples, n = 36 blank/control samples) produced 12,968,125 16 S rRNA sequence reads, of which 9,920,805 reads were retained after quality control (77%). A total of 8,974,393 sequences, with a mean read count of 249,288 reads per sample, were retained after the sequences corresponding to the blank control samples were removed. After rarefying to 95,870 sequences per sample, *de novo* OTU picking returned 228 OTUs, of which 19 OTUs corresponding to the mock community make up more than 99% of the pooled community. The average error rate of the run was found to be 0.040% (±0.004).

### Effect of different amplification method

**Figure 2:**
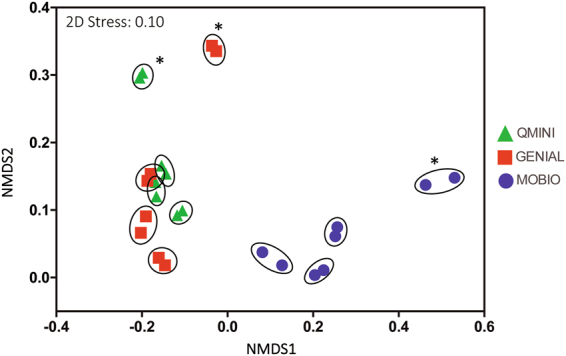
Clustering of samples amplified with two different polymerases on a non-metric multidimensional scaling (NMDS) plot of the Bray-Curtis dissimilarities. Points are colored by applied extraction kit. The encircled pairs correspond to a single sample where each data point represents one 16 S rRNA amplification with Phusion Hot Start II High-Fidelity DNA Polymerase and the another with Platinum SuperFi DNA Polymerase. Sample pairs labeled with * were stored in PBS.

The choice of polymerase (Platinum SuperFi DNA polymerase vs. Phusion Hot Start II High-Fidelity DNA polymerase) was not found to significantly change the number of observed OTUs (p = 0.08 [paired t-test] or Inverse Simpson index, p = 0.48, [paired t-test]). Furthermore, pairwise comparison of the Bray-Curtis dissimilarity between the two polymerases yielded only small variations (maximum difference 0.076, Supplementary Table S2) indicating near identical bacterial community profile for a single sample (Fig. 2). Since the results indicate that these two applied high-fidelity polymerases do not significantly impact the observed microbial diversity, we pooled the data from the two polymerases for identical sample for the analyses of buffer and extraction kit choice. The addition of five cycles in 16 S rRNA gene amplification shows only a minor impact on the detected bacterial composition when tested on MOBIO extractions (Supplementary Fig. S2a). There was, however, a significant increase of the number of OTUs detected with additional cycles (p = 0.029, Supplementary Fig. S2b), indicating that lower cycle numbers are favorable.

### Effect of storage buffer

**Figure 3:**
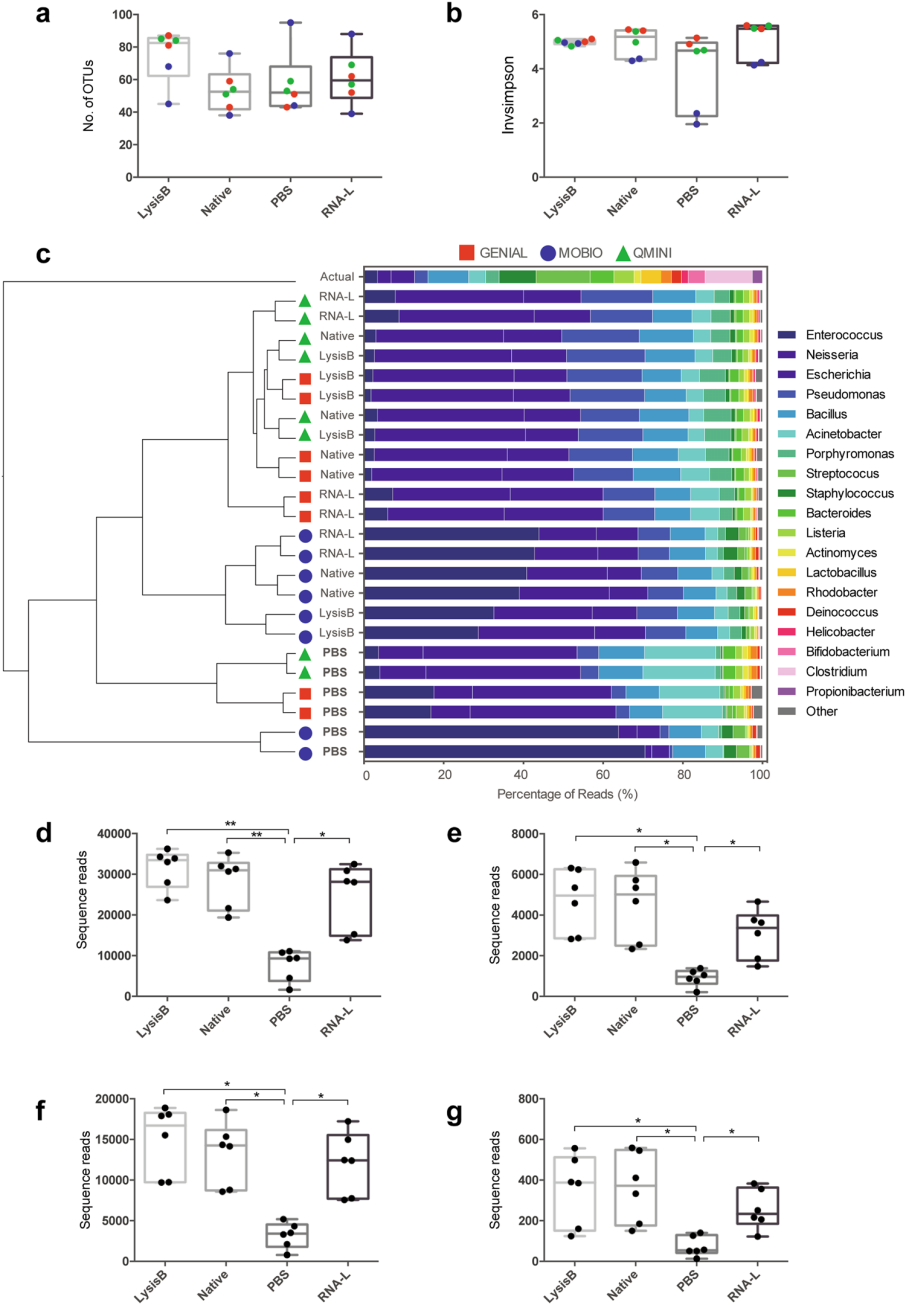
PBS stored samples significantly distort individual OTUs and cluster separately from other buffer types. Boxplots (median ± range) of (**a**) the number of OTUs and (**b**) the Inverse of the Simpson index for each buffer type. (**c**) UPGMA clustering on Bray-Curtis dissimilarities including taxa plots showing the relative abundance of OTUs in percentage of reads. Differential clustering of PBS to all other buffers was found to be significant (parsimony test, p = 0.001) (**d**–**g**) Individual bacterial OTUs are significantly underrepresented for PBS-stored samples. Number of sequence reads for OTUs corresponding to (**d**) *Neisseria*, (**e**) *Pseudomonas*, (**f**) *Porphyromonas*, and (**g**) *Helicobacter*. (Wilcoxon test, *p < 0.05, **p < 0.01).

The effect of the four storage buffer (lysis buffer, native, PBS or RNA-later) on the alpha diversity was assessed based on OTU richness (identified absolute number of taxa) and evenness (Inverse Simpson index). The choice of storage buffer had no significant influence on the OTU richness of the swab samples (p = 0.158 [ANOVA], Fig. 3a), nor the overall evenness. However, PBS treated samples that were extracted with MOBIO, detected a lower evenness compared to all other treatment conditions (Wilcoxon test, Fig. 3b).

Pairwise AMOVA of Bray-Curtis dissimilarity showed that the storage buffer choice had a significant impact on the community structure (p = 0.004, AMOVA). A dendrogram of the Bray-Curtis dissimilarity shows that the PBS stored samples clustered separately from the other buffer types which was confirmed by parsimony analysis (p = 0.001, Fig. 3c). To examine which OTUs drive the differential clustering, we examined the read count for each OTU. Four bacterial OTUs corresponding to *Neisseria*, *Pseudomonas*, *Porphyromonas* and *Helicobacter* are significantly different in the PBS stored samples for all extraction kits (Fig. 3d–g). These results indicated that PBS buffer significantly alters single OTUs as well as the overall bacterial composition compared to all other storage buffers, independent of extraction kit choice. The bacterial profile of the blank control samples indicated that this effect is not caused by a buffer specific contamination as there appears to be no obvious buffer or kit specific profile (Supplementary Fig. S3).

### Effect of extraction method

Richness, both the observed number of OTUs and Choa1, were analyzed to see the effect of the extraction kit choice on the alpha diversity. Pairwise comparison showed no significant effect on OTU richness between the different extraction kits (p = 0.893 [ANOVA], Table 2). In general, all extraction kits detect a higher OTU richness compared to the expected richness of the mock community (Table 2). In addition to assessing richness, evenness was analyzed using the Inverse Simpson index. The evenness of the samples extracted using MOBIO was significantly lower compared to the QMINI and GEN-IAL extractions (p = 0.008, p = 0.023, Wilcoxon test, Table 2). The evenness did not significantly vary between QMINI and GEN-IAL. Yet, the mean (±SEM) observed evenness (5.21 ± 0.08) was significantly lower than the expected evenness of the mock community (18.3). The same five OTUs, *Enterococcus, Neisseria, Escherichia, Pseudomonas*, and *Bacillus* dominate the bacterial profile independent of extraction kit choice (Fig. 3c).

**Table 2:** Alpha diversity measurements (mean ± SEM) for each of the DNA extraction kits (n = 8).

Extraction Method	Observed OTUs	Chao1	InvSimpson
MOBIO PowerLyzer PowerSoil Kit	62.88 ± 8.38	69.79 ± 10.31	3.9 ± 0.40
GEN-IAL First All-Tissue Kit	59.75 ± 5.82	66.02 ± 7.51	5.3 ± 0.09
QIAamp DNA Mini Kit	64.00 ± 4.87	78.04 ± 5.94	5.1 ± 0.13
Actual/Expected Mock Community	22	22	18.3

**Figure 4:**
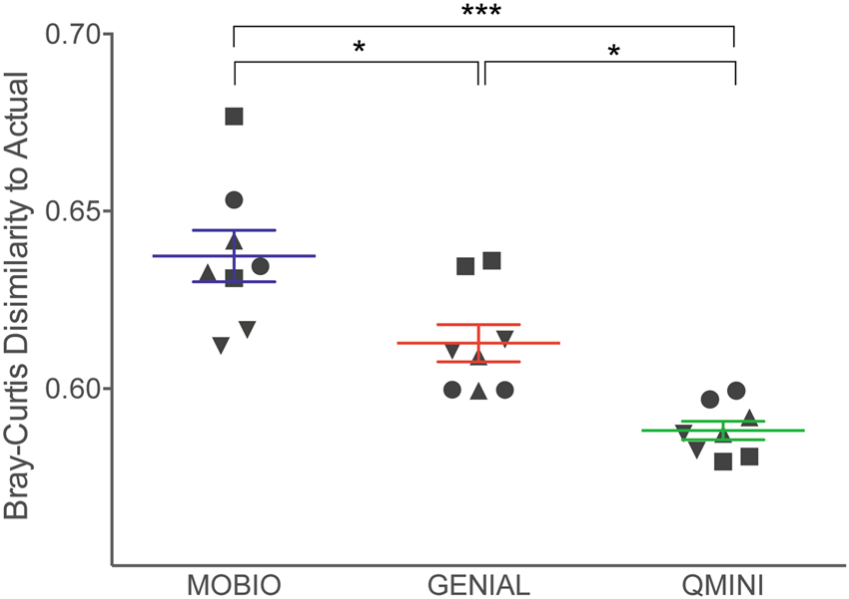
Bray-Curtis dissimilarity between observed and expected strain proportion for each of the tested extraction methods. The expected strain proportion (actual) was generated for comparison and represents the theoretically composition of the mock community (see methods for detail). The pair-wise proportions (expected to observed) from samples extracted with the same commercial kit were grouped in a single boxplot (mean ± SEM). Symbols illustrate different buffer types (■ PBS, ▼RNA-later, ▲native, ●lysis buffer) (Paired t-test, *p < 0.05, ***p < 0.001).

Pairwise AMOVA of Bray-Curtis dissimilarity indicated that the extraction kit choice significantly impacted the community structure (p = 0.001, AMOVA). To assess which extraction kit more accurately represents the bacterial community structure, a theoretical ideal mock community (actual) composition was created for comparison (see methods for details). In the ideal scenario, the experimental data would be identical to the actual composition and there would be no Bray-Curtis dissimilarity. To assess the extraction kits, Bray-Curtis dissimilarity was calculated between the observed and actual mock community for each sample (Fig. 4). The samples extracted with the same commercial kit were grouped in a boxplot and pairwise comparison was performed. The QMINI kit produced a significantly better representation of the bacterial community compared to all other kits tested (paired t-test, all p < 0.01, Fig. 4). On the contrary, the MOBIO kit performed significantly poorer than all other tested kits (all p < 0.01, Fig. 4). Overall, all the extraction kits distort the bacterial profile compared to the expected bacterial composition of the mock community (Fig. 4).

## Discussion

We compared a variety of storage buffers, extraction kits, and amplification methods to examine which combination of handling conditions best represents the microbial diversity of an even mock community (Fig. 1). Different combinations of factors that most closely resemble the reality of sample handling were analyzed to facilitates the establishment of standards for the analyses of microbial compositions in swab samples. We show that the choice of storage buffer and extraction kit affects the detected bacterial composition, while different amplification methods had only a minor effect.

Using a mock community, four storage buffers were tested that have been previously used in various studies^25–28^. All samples in this study were frozen at −80 °C rapidly after collection. The samples stored in RNA-later, lysis buffer and native performed similarly to each other and revealed a similar detected bacterial diversity (Fig. 3). Samples stored in RNA-later have been previously reported to decrease DNA purity, lower DNA extraction yields, and to significantly alter the microbial diversity compared to native frozen samples^10,29^. This, however, was not observed in our study. It is likely, that this reflects differences in the sample material (microbes on swab vs. fecal samples) as it has been observed that fecal samples are harder to disperse evenly in RNA-later which may affect the storage and extraction efficiency^10^. Interestingly, compared to the other buffer types, swabs stored in PBS show an altered bacterial composition. There is no indication of a PBS buffer specific contamination profile in the blank samples that could explain this differential clustering. Moreover, PBS buffer in combination with the MOBIO extraction kit detected a lower evenness, which indicates that PBS seems to be particularly incompatible with certain extraction kits. PBS is a balanced salt solution that maintains pH, osmotic balance and is therefore frequently used as a wash buffer in cell and tissue culture. PBS storage has been recommended by manufacturers protocols and has been previously used when examining various extraction kits^12,30^. Other studies examining the effect of different storage conditions have not tested PBS despite its use in DNA extraction from swab material^6,10–12^. It is not clear what properties of PBS effect the mock community differently from other storage buffers. Due to its properties, the buffer may stabilize certain cell types and therefore create a different bacterial profile. Interestingly, despite the different bacterial profile, the PBS samples perform similarly to the other buffer types when comparing them to the mock community. This indicates that the choice of buffer can affect the bacterial profile and specific OTUs, but does not lead to a significantly worse representation of the bacterial community. Our findings support the notion that standardization in sample collection and handling is essential to allow comparison of data within a study^31^. Additionally, field-wide standardization across handling protocols is vital for each sample type, so that cross-study comparisons become possible.

All extraction methods used in this study identify all 19 OTUs present in the mock microbial community (22 bacterial strains of 19 genera, Supplement Table S1). However, all kits detected a higher richness compared to the actual richness of the mock control. A low concentration of mock community (approximately 1 × 10^7^ cells/mL of each organism) was used in this study to simulate the expected bacterial amount in vaginal or oral swab samples^32^. Therefore, it was not surprising that additional OTUs were detected^13^. However, 99% of the pooled library clusters into 19 OTUs which correspond to the bacteria in the mock community. This indicates that the additionally detected OTUs correspond to a small fraction of sequence reads and may therefore be a result of contamination. This study in combination with previous work suggests that the expected biomass of vaginal and oral swab samples is sufficient for amplicon-based microbial detection without the need of additional target enrichment^13^. The use of a mock microbial community in this study allowed for direct assessment of the extraction kit performance. This comparison indicated that QMINI provides the best representation of the bacterial community when compared to MOBIO and GENIAL. Using a mock community, Yuan *et al*. also found that an altered version of QMINI provided the best bacterial profile^6^. A study using oral swabs confirmed that QMINI extracts DNA with significantly greater yield and good quality compared to other extraction kits^2^. This is in contrast to previous studies on fecal and soil samples, which found that MOBIO most effectively extracts microbial DNA of various bacterial strains^33^. These reported differences in optimal extraction kit may be due to the differences in sample type. The overall bacterial DNA and exogenous material (e.g. fiber) differs substantially between fecal and swab material^34^. Standardization of the extraction kit may thus only be appropriate within each sample type.

In this study, we find that the choice between the two polymerases and the addition of five cycles in amplification of the 16S rRNA gene did not have a significant effect on the bacterial community structure (Fig. 2). Contrary to our findings, Wu *et al*. report that the choice of polymerase had an effect on the microbial community structure, however, the two polymerases that were tested had considerable differences in the fidelity (20 times and 4 times higher than *Taq*)^35^. The two hot-start polymerases used in our study, had significantly higher fidelity (100 times and 52 times higher compared to *Taq*) and are both recommended for NGS applications by the manufacturers. This may likely explain the lack of observable differences. Unlike polymerase choice, which had no effect on the detected evenness or richness, the addition of five PCR cycles to the amplification method led to an overestimation of the bacterial richness. Previous studies have already suggested that this increase is due to an upsurge of chimeric structures with increased cycle numbers^3,5,35^. This supports the notion that lower cycles numbers are favorable for amplicon sequencing^5^.

All tested conditions in this study lead to a distortion of the bacterial community structure compared to the expected bacterial mock composition (Fig. 4). *Enterococcus, Neisseria, Escherichia*, and *Pseudomonas* dominated the detected profile in our study, while other bacteria genera such as *Lactobacillus* were underrepresented. Knowledge of which genera are underestimated in the detected bacterial profile (e.g. *Lactobacillus*) is essential to properly estimate the bias when studying certain bacterial communities (e.g., the vaginal microbiome). In a recent study using the same mock community, the bacterial profile resembled the one detected in our study, indicating that the observed distortion is most likely not due to laboratory or kit specific contamination^3,13^. Instead, the bias could be attributed to a variety of factors that were not examined in this study, such as differential susceptibility of bacteria to lysis^6^. To increase lysis efficiency of a broader spectrum of bacteria, enzymatic pre-treatment has been studied as a potential solution, with mixed results^6,36,37^. Another potential cause for the observed bias is the use of primers for 16 S rRNA gene amplification. Although these are universal, amplification may favour certain bacterial strains thus creating bias in the analysis^38,39^. Shotgun metagenomics has been proposed as a solution as it negates some of the bias caused by the amplification, however, this technique does not negate all of technical caveats as storage and extraction kit choice can still have a major impact on the results^3,40^. Continual improvement to the sample handling conditions for both amplicon sequencing and shotgun metagenomics using mock communities is therefore essential.

## Conclusion

For now, investigators should standardize the sample handling methods for each sample type as consistency among sample collection, sample storage and sample processing is able to significantly reduce variation. Preliminary tests on specific sample types should be used to ensure that the comparative analysis is as accurate as possible. Caution is, however, warranted when drawing conclusions about the relative abundance of bacterial populations in a single sample and when combining data for meta-analyses.

## Electronic supplementary material

Supplementary Information
