# Supplementary material for: The impact of storage buffer, DNA extraction method, and polymerase on microbial analysis
Source: Sci Rep. 2018 Apr 19;8:6292. doi: 10.1038/s41598-018-24573-y (PMC5908915; doi:10.1038/s41598-018-24573-y)
Supplement: Supplementary file 1 — Supplementary Information [file 41598_2018_24573_MOESM1_ESM.pdf]

The impact of storage buffer, DNA extraction method, and polymerase on microbial analysis

Luisa K Hallmaier-Wacker[1,2], Simone Lueert[1,2], Christian Roos,[2] Sascha Knauf,[1*]

Supplementary Information:

Table S1: Organisms in the microbial mock community HM-280

Organism	16S rRNA gene copy number
Acinetobacter baumannii, strain 5377	5
Actinomyces odontolyticus, strain 1A.21	2
Bacillus cereus, strain NRS 248	12
Bacteroides vulgatus, strain NTC 11154	7
Bifidobacterium adolescentis, strain E194a	5
Clostridium beijerinckii, strain NCIMB 8052	14
Deinococcus radiodurans, strain R1 (smooth)	3
Enterococcus faecalis, strain OG1RF	4
Escherichia coli, strain MG1655	7
Helicobacter pylori, strain 26695	2
Lactobacillus gasseri, strain 63AM	6
Listeria monocytogenes, strain EGDe	6
Neisseria meningitides, strain MC58	4
Porphyromonas gingivalis, strain 2561	4
Propionibacterium acnes, strain KPA171202	3
Pseudomonas aeruginosa, strain PAO1-LAC	4
Rhodobacter sphaeroides, strain ATH 2.4.1	3
Staphylococcus aureus, strain TCH959	6
Staphylococcus epidermidis, FDA strain PCI 1200	5
Streptococcus agalactiae, strain 2603 V/R	7
Streptococcus mutans, strain UA159	5
Streptococcus pneumoniae, strain TIGR4	4

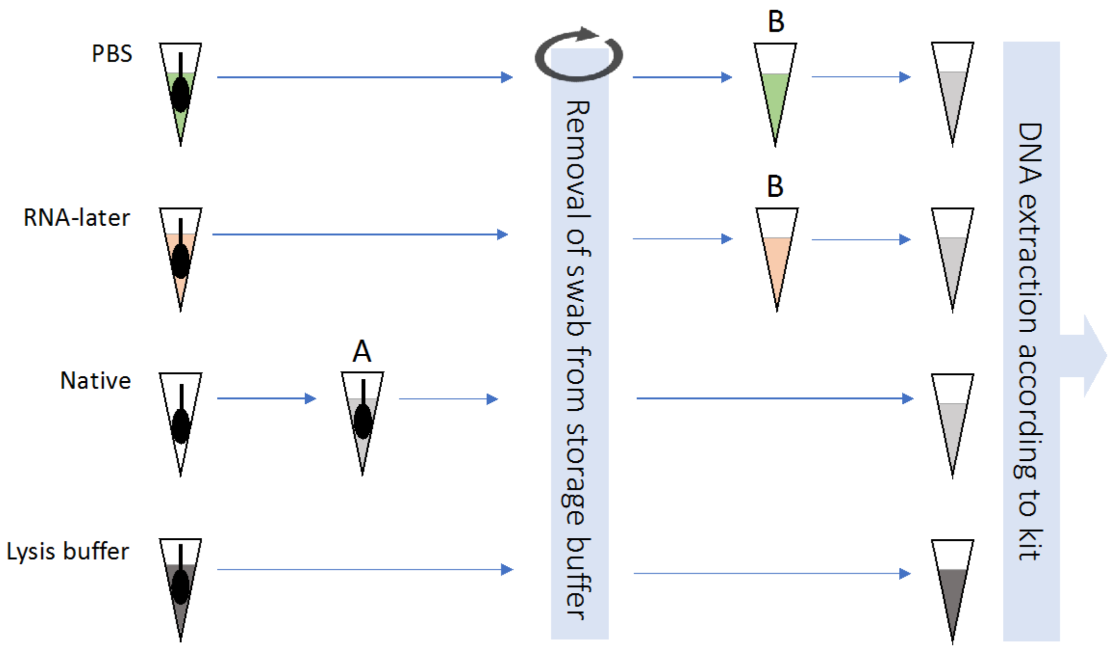

Figure S1: Processing of swab samples prior to DNA extraction. (A) A kit-specific lysis buffer was added to the native swab sample, which was briefly vortexed to ensure even distribution of the buffer. Subsequently, the bottom of the swab-containing tubes (PBS, RNA-later, native and custom-made lysis buffer) was pierced by a sterile needle (20G) to allow for the separation of the swab material from the respective storage buffer. Separation was forced by centrifugation (2,500g for 1 minute at room temperature). (B) Prior to DNA extraction, RNA-later and PBS buffer were centrifuged at 11,000g for 5 minutes at 4 °C. The supernatant was removed and replaced by kit-specific lysis buffer.

Table S2: Bray-Curtis Dissimilarity between identical samples amplified with either Platinum SuperFi DNA Polymerase or the Phusion Hot Start II High-Fidelity DNA Polymerase.

Kit	Buffer	Bay-Curtis Dissimilarity
MOBIO	Lysis buffer	0.064
	Native	0.039
	PBS	0.076
	RNA-later	0.032
GENIAL	Lysis buffer	0.028
	Native	0.036
	PBS	0.027
	RNA-later	0.025
QMINI	Lysis buffer	0.062
	Native	0.076
	PBS	0.019
	RNA-later	0.044

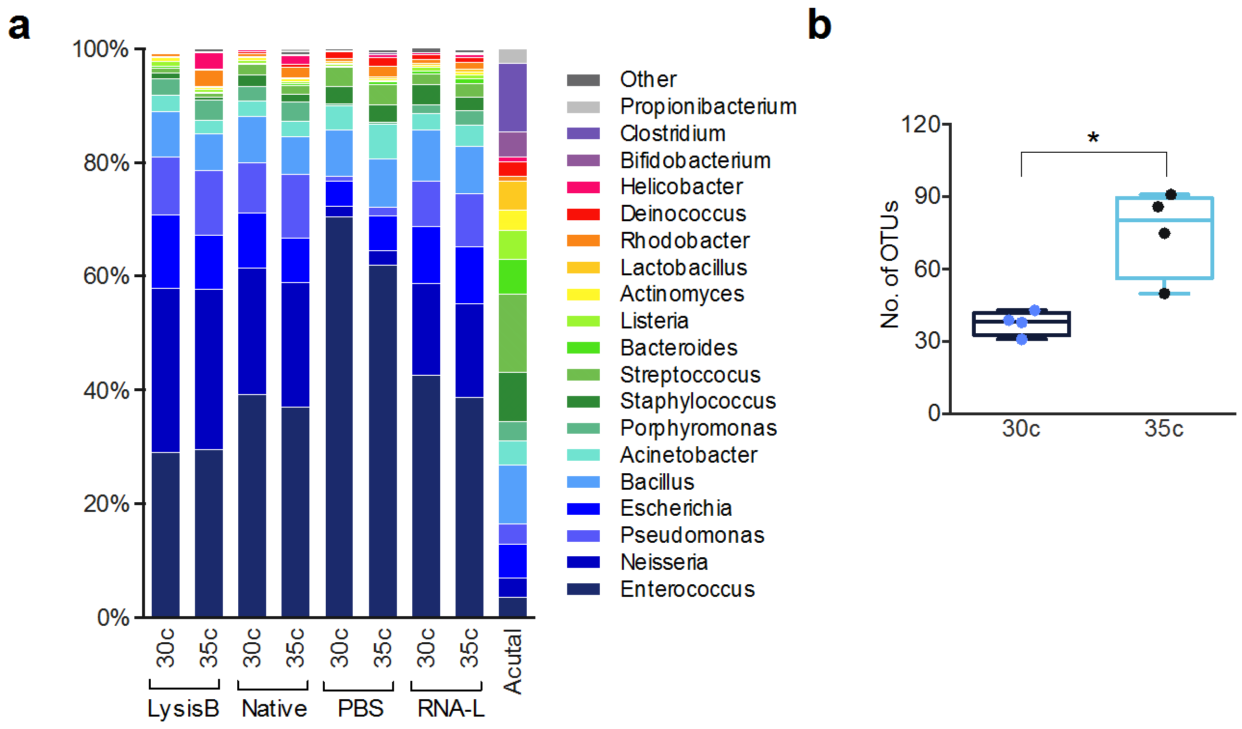

Figure S2: **Five additional cycles ('c') significantly altered the number of detected OTUs but did not alter the bacterial composition**. (a) Taxa plots showing the relative abundance of OTUs identified in percentage of reads. Actual refers to the predicted bacterial composition of the mock community. (b) Boxplots (median ± range) of the number of OTUs in each of the samples plotted by cycle number ('c'). (Wilcoxon t-test, *p<0.05)

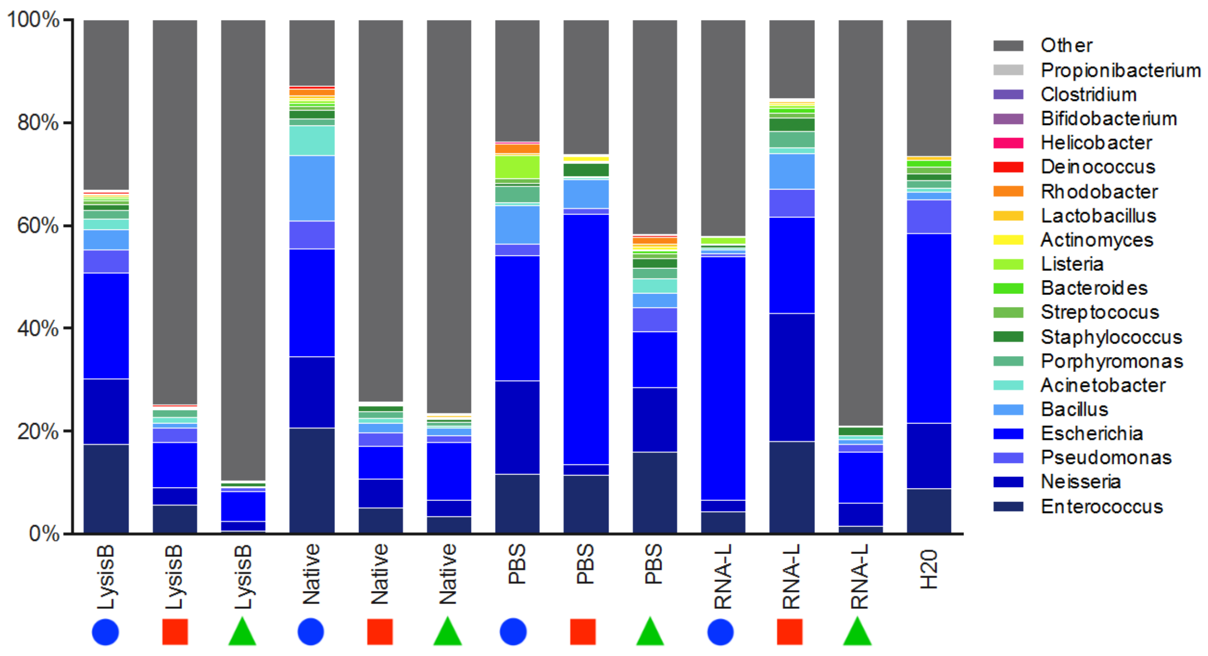

Figure S3: Bacterial profile of blank samples for each buffer and kit used in our study. A total of 946,412 sequences corresponded to the blank sample, with a mean read count of 12,727 reads per sample. Taxa plots showing the relative abundance of OTUs identified in percentage of reads. Symbols illustrate different extraction kits (●MOBIO, ■ GENIAL, ▲QMINI)
